# Longitudinal analysis of culture of patient safety survey results in surgical departments

**DOI:** 10.3389/frhs.2024.1419248

**Published:** 2024-10-24

**Authors:** Logan R. Butler, Shaian Lashani, Cody Mitchell, Jin H. Ra, Caprice Greenberg, Lawrence B. Marks, Thomas Ivester, Lukasz Mazur

**Affiliations:** ^1^ Division of Healthcare Engineering, School of Medicine, University of North Carolina at Chapel Hill, Chapel Hill, NC, United States; ^2^ Division of Acute Care Surgery, Department of Surgery, School of Medicine, University of North Carolina at Chapel Hill, Chapel Hill, NC, United States; ^3^ Department of Surgery, School of Medicine, University of North Carolina at Chapel Hill, Chapel Hill, NC, United States; ^4^ Department of Radiation Oncology, School of Medicine, University of North Carolina at Chapel Hill, Chapel Hill, NC, United States; ^5^ UNC Health, University of North Carolina at Chapel Hill, Chapel Hill, NC, United States; ^6^ School of Information and Library Science, University of North Carolina at Chapel Hill, Chapel Hill, NC, United States

**Keywords:** patient safety, safety culture, surgery, AHRQ, SOPS

## Abstract

**Background:**

There is a need for improved methodologies on how to longitudinally analyze, interpret and learn from the Surveys on Patient Safety Culture™ (SOPS), developed by the Agency for Healthcare Research and Quality (AHRQ). Typically, SOPS quantify results by the percentage of positive responses, but this approach may miss insights from neutral or negative feedback.

**Study design:**

The SOPS were distributed every two years from 2011 to 2022 to all hospital staff at one academic institution from perioperative services. Differences between rates of “positive” and “negative” scores (“Delta”), and “neutral” responses over time were calculated. The coefficient of determination (*R*^2^) was used to assess the correlation strength of the positive scores as the primary outcomes provided by the SOPS and Delta values over time. Finally, we evaluated patterns (crossing and converging [indicating “worrisome” patterns] vs. diverging [suggesting “desirable” pattern] vs. stable [suggesting “neutral” pattern]) of the longitudinal scores.

**Results:**

A total of 1,035 responses were analyzed [51 and 40 survey items for SOPS v1 and v2 (2022 only), respectively]. Comparing the *R*^2^ values of the positive only scores to the Delta scores demonstrated a change in effect size for “Nonpunitive Response to Error” (*R*^2^ = 0.290 vs. 0.420). Of the 13 specific categories measured through SOPS, plotting negative vs. positive values elucidated 2 crossing, 2 converging and 2 diverging patterns indicating both a decrease in positive responses and an increase in negative responses rather than neutral.

**Conclusion:**

Longitudinal analysis of the SOPS using the directional measures, Delta and pattern trends can provide organizations with additional key insights regarding culture of patient safety.

## Introduction

Safety culture refers to the shared values, beliefs, and norms within an organization that influence behaviors and promote safety. In the context of healthcare, it encompasses the collective understanding of what is important regarding patient safety, as well as how systems operate within a healthcare organization (1–3). Patient safety culture is recognized as a crucial element in establishing and implementing patient safety programs. A few reasons that this is important is because patient safety culture assessment and improvement promote better patient outcomes, improved staff well-being, reductions in medical errors, and enhanced organizational effectiveness which can be appreciated on a global scale (4). Efforts to improve patient safety often prioritize the development of a strong safety culture within healthcare organizations. Some variables that define patient safety culture include overall patient safety perception, communication, leadership support, teamwork, staffing, and event reporting and analysis (5, 6). The fluid concept of safety culture can be difficult to measure as safety culture is defined based on a variety of variables, however, it has been demonstrated to be predictive of future performance across several accounts. Surgical teams have shown categorical and quantitative improvements after implementing tools like operating room checklists to help prepare for surgery, remember important information, and ensure medication safety (7–9). Overall, prior research highlights the importance of fostering a culture of safety using mechanisms such as event reporting, analysis, and continuous quality improvement efforts and how these actions contribute to attitudes of patient safety within hospital departments.

Within healthcare, the Agency for Healthcare Research and Quality's (AHRQ) Surveys on Patient Safety Culture™ (SOPS) is a commonly used tool to assess safety culture using questions to measure the functional categories of communication, teamwork, leadership, and overall perceptions of safety. Traditionally, the method of quantifying the results from the AHRQ survey is to assess the percent of positive responses. This conventional method may be suboptimal because it overlooks valuable insights from employees who perceive the safety culture negatively, remain neutral, or whose responses fluctuate over time. Nevertheless, past studies have shown the reliability, predictive validity, and psychometric soundness of the SOPS using surveys from hundreds of hospitals and found that it was reliable, valid, and psychometrically sound to use as an assessment tool for patient safety culture analysis (10–12).

We herein present an alternative approach to quantify the AHRQ survey that considers both the percent positive, and the percent negative, responses. Data gathered through the completion of the SOPS includes evaluations of overall patient safety perceptions and views on communication, leadership, and teamwork from the perspective of the healthcare personnel completing the survey. The intention of gathering this data is to demonstrate targetable areas for improvement within specific populations. This manuscript aims to explain an innovative method of analysis and the conclusions that can be drawn from the AHRQ SOPS data gathered over several years at a large academic institution within surgical departments. Ultimately, these conclusions and analyses could be used to understand the common problems within a large healthcare setting and assist in designing subsequent interventions to improve culture and patient safety outcomes.

## Methods

Our institution distributed the AHRQ SOPS to all departments every two years from 2011 to 2019 and in 2022. Survey participation is voluntary, and responses are collected anonymously. Data analyses were conducted between December 2022 and April 2023. These six surveys over 11 years were filtered to include only the results collected from the 20 surgical departments/units/service lines representing surgical services. The surgical departments/units/service lines, provider roles, and work areas were used to select the data that can be seen in Table 1. The AHRQ made changes to the survey questions in the 2022 iteration of the SOPS (transition from SOPS v1 to v2), including removing individual questions, rewording questions, and relabeling result categories (13). Specifically, the final SOPS 2.0 has 40 survey items compared with 51 survey items in SOPS 1.0. The names of some composite measures were changed to align with changes to the content assessed in those measures. To compare the results of the 2022 survey to the surveys conducted between 2011–2019, individual questions for each survey in each category were matched to ensure analogous questions were compared across all surveys. The matched specific categories across surveys can be seen in Table 2. For each specific category included in the SOPS, the individual questions used to generate each category score each year were paired with the matching questions across all surveys. The percentage of positive responses (“Strongly Agree” or “Agree”) and negative responses (“Strongly Disagree” or “Disagree”) were calculated for each question and compared between years. The difference between the percentage positive and negative responses (Delta) was also calculated to compare shifts in the cultural perceptions of the survey population.

**Table 1:** Data stratification of operating room departments from the AHRQ HSCS.

Filter label	Order of sorting	Included filters
By department/unit/service line	First	Operating Room, Neurosurgery Operating Room, Neurosurgery Anesthesiology Support, Neurosurgery Anesthesiology Department, PCS/PACU Main, Anesthesia Support, Anesthesiology Department, Central Sterile Supply, GI Procedures, Transplant Program, Trauma Program, Children's Operating Room, Operating Room—Main, Children's PCS/PACU, Surgical Support Services, Attending Physicians, Resident Physicians, PACU Main, Department of Neurosurgery—MD participants, Department of Orthopaedics—MD participants, Department of Surgery—MD participants, and Central Distribution
By role	Second	Advanced Practice Nurse (NP, CRNA, CNS, CNM); Registered Nurse (RN); Physician Assistant; Resident, Intern; Physician, Attending, Hospitalist; Pharmacist, Pharmacy Technician; and Supervisor, Manager, Department Manager, Clinical Leader, Administrator, or Director
By work area	Third	Combined Medical/Surgical Unit; Surgical Unit; Anesthesiology; Pre-Op, Operating Room/Suite, PACU/Post Op, Peri Op

**Table 2:** AHRQ SOPS questions categories and corresponding letter (A-N) legend.

	Question categories		
	Specific categories (2022)	Specific categories (2019–2011)	Letter legend
Summary	Overall safety score	Overall safety score	A
Overall Perceptions	Patient safety rating	Unit grade	B
	–	Perceptions of safety	C
Communication	Communication about error	Nonpunitive response to error	D
	Communication openness	Communication openness	E
	Response to error	Feedback about error	F
	Most employees report events	Most employees report events	G
	Reporting events	Error reporting frequency	H
Teamwork	Teamwork	Teamwork across and within units (Composite)	I
	Handoffs	Handoffs	J
Leadership	Management support for safety	Management support for safety	K
	Supervisor support or safety	Supervisor support for safety	L
	Staffing and workplace	Staffing	M
	Learning from errors	Learning from errors	N

The SOPS results were calculated by averaging the percentage of positive responses for each question within a category to generate a category score. To visually compare changes in reported SOPS results over time, these results were plotted by year to determine longitudinal changes. Additionally, the delta scores for each category were analyzed to determine the pattern (crossing and converging [indicating “worrisome” patterns] vs. diverging [suggesting “desirable” pattern] vs. stable [suggesting “neutral” pattern]) in positive vs. negative perceptions of survey respondents over time. Specifically, a crossing pattern implies that there is a change in direction of responses over time (if positive responses decrease while negative responses increase); a converging pattern suggests that positive and negative responses are moving closer together; a diverging response indicates that positive and negative responses are moving further apart; a stable pattern indicates a consistent perception. We sought to determine which domains within the SOPS showed the greatest change over time (in either the positive or negative direction) to inform design of interventions that will have the greatest impact on patient safety moving forward.

To compare the rate of change over all years analyzed in scores for each category, linear regression and the coefficient of determination (R^2^) were determined for each category. Utilizing the absolute R-squared values, the strength of a relationship for each value was determined using the following generalized strength categories: R-squared value <0.3 is generally considered a None or Very Weak effect size, an R-squared value 0.3 ≤ *r* < 0.5 is generally considered a Weak or Low effect size, a value 0.5 ≤ *r* < 0.7 is generally considered a Moderate effect size, and a value *r* ≥ 0.7 this value is generally considered Strong effect size (14, 15).

## Results

Positive, negative and delta values over time are shown in Table 3. Linear regression coefficients and *R*^2^ values for the positive-only and calculated Delta scores and corresponding patterns of the positive vs. negative responses over time are displayed in Table 4. For both analyses, the categories with the greatest declines in reported safety culture scores were “Patient Safety Rating/Unit Grade”, “Management Support for Safety”, “Learning from Errors”, and “Staffing”. The most improvement over the same period was seen in the category “Most Employees Report Events” in both analyses. Overall, *R*^2^ values were only different in one category (“Nonpunitive Response to Error”), with positive vs. Delta results of 0.29 vs. 0.41, respectively.

**Table 3:** Positive, negative, and delta results over time.

		Sum-mary	Overall perceptions		Communication					Teamwork		Leadership				*N*
		A	B	C	D	E	F	G	H	I	J	K	L	M	N	
2011	% Positive responses:	59.7	72.1	58.8	40.0	59.8	55.5	57.1	59.3	65.8	43.6	64.7	75.0	56.5	71.3	183
	% Negative responses:	20.4	5.81	23.3	34.8	12.8	17.5	42.8	13.9	19.7	31.1	19.5	12.9	27.2	12.4	
	Delta	39.2	66.2	35.4	5.23	46.9	38.0	14.2	45.3	46.1	12.5	45.2	62.1	29.3	58.8	
2013	% Positive responses:	55.3	62.6	51.4	38.1	58.9	57.5	54.1	47.0	61.6	37.3	59.1	68.6	59.6	62.9	221
	% Negative responses:	22.1	9.8	30.2	35.6	12.7	14.4	45.8	20.8	19.8	36.9	21.9	15.7	23.6	14.0	
	Delta (%)	33.1	52.8	21.2	2.58	46.2	43.1	8.26	26.2	41.7	0.36	37.1	52.8	36.0	48.9	
2015	% Positive responses:	50.7	54.7	46.5	37.5	51.1	42.4	64.3	43.0	60.4	33.2	51.8	70.0	48.1	63.6	133
	% Negative responses:	24.6	16.6	32.7	37.4	12.8	23.7	35.6	20.4	20.3	38.1	24.7	15.5	34.3	14.5	
	Delta (%)	26.1	38.1	13.7	0.130	38.2	18.6	28.6	22.5	40.1	−4.94	27.0	54.5	13.8	49.0	
2017	% Positive responses:	58.4	69.3	61.1	41.1	63.4	57.0	66.4	49.9	65.7	36.9	61.0	78.8	48.5	71.5	158
	% Negative responses:	17.6	6.00	19.6	33.8	11.5	12.6	33.5	13.1	12.6	30.4	17.1	8.00	32.9	7.42	
	Delta (%)	40.7	63.3	41.4	7.29	51.8	44.4	32.9	36.8	53.0	6.50	43.9	70.8	15.5	64.1	
2019	% Positive responses:	53.3	62.3	52.3	37.9	59.8	47.8	79.7	50.0	62.9	33.5	53.3	73.2	38.7	67.4	163
	% Negative responses:	22.6	9.26	29.1	34.5	10.8	16.8	20.2	17.0	15.6	37.1	23.1	13.7	44.9	13.1	
	Delta (%)	30.7	53.0	23.1	3.44	48.9	31.0	59.5	33.0	47.3	−3.58	30.1	59.4	−6.21	54.3	
2022	% Positive responses:	46.7	32.2	–	45.6	51.9	44.8	74.0	48.4	65.6	44.8	32.3	62.7	23.0	47.8	177
	% Negative responses:	24.7	24.2	–	17.5	12.8	26.0	25.9	12.1	17.7	18.3	43.1	18.0	58.6	23.3	
	Delta (%)	21.94	7.91	–	28.12	39.12	18.81	48.02	36.23	47.87	26.47	−10.78	44.63	−35.56	24.48	

**Table 4:** Coefficients of determinations (*R*^2^) for positive scores vs. delta values and emerging patterns.

		Positive SOPS scores		Delta (% Positive −% Negative) scores		Patterns
SOPS category	Specific category using SOPS 2.0 categories	Slope (Change in score/year)	*R* ^2^	Slope	*R* ^2^	Crossing vs. Convergent vs. Divergent vs. Stable
Summary	Overall safety score	−1.80	0.482	−2.26	0.335	Divergent
	Patient safety rating	−5.30	0.474	−7.59	0.435	Divergent
Communication	Response to error	0.889	0.295	3.54	0.419	Convergent
	Communication openness	−0.691	0.070	−0.488	0.028	Stable
	Feedback about error	−1.93	0.296	−3.04	0.243	Stable
	Most employees report events	4.67	0.801	9.33	0.801	Convergent
	Reporting events	−1.10	0.146	−0.317	0.005	Stable
Teamwork	Teamwork	0.2414	0.036	1.1061	0.197	Stable
	Handoffs	−0.0500	0.0004	1.98	0.097	Stable
Leadership	Management support for safety	−4.86	0.621	−8.11	0.538	Crossing
	Supervisor support for safety	−1.11	0.138	−1.46	0.094	Stable
	Staffing and workplace	−6.57	0.851	−12.8	0.842	Crossing
	Learning from errors	−2.74	0.342	−4.01	0.297	Stable

Crossing: the proportion of negative responses overtook the proportion of positive responses over time; Converging: the proportions of negative and positive responses drew closer to being equal over time; Diverging: the proportions of negative and positive responses are drew further from being equal over time; Stable: the proportions of negative and positive responses remained relatively the same over time.

**Figure 1:**
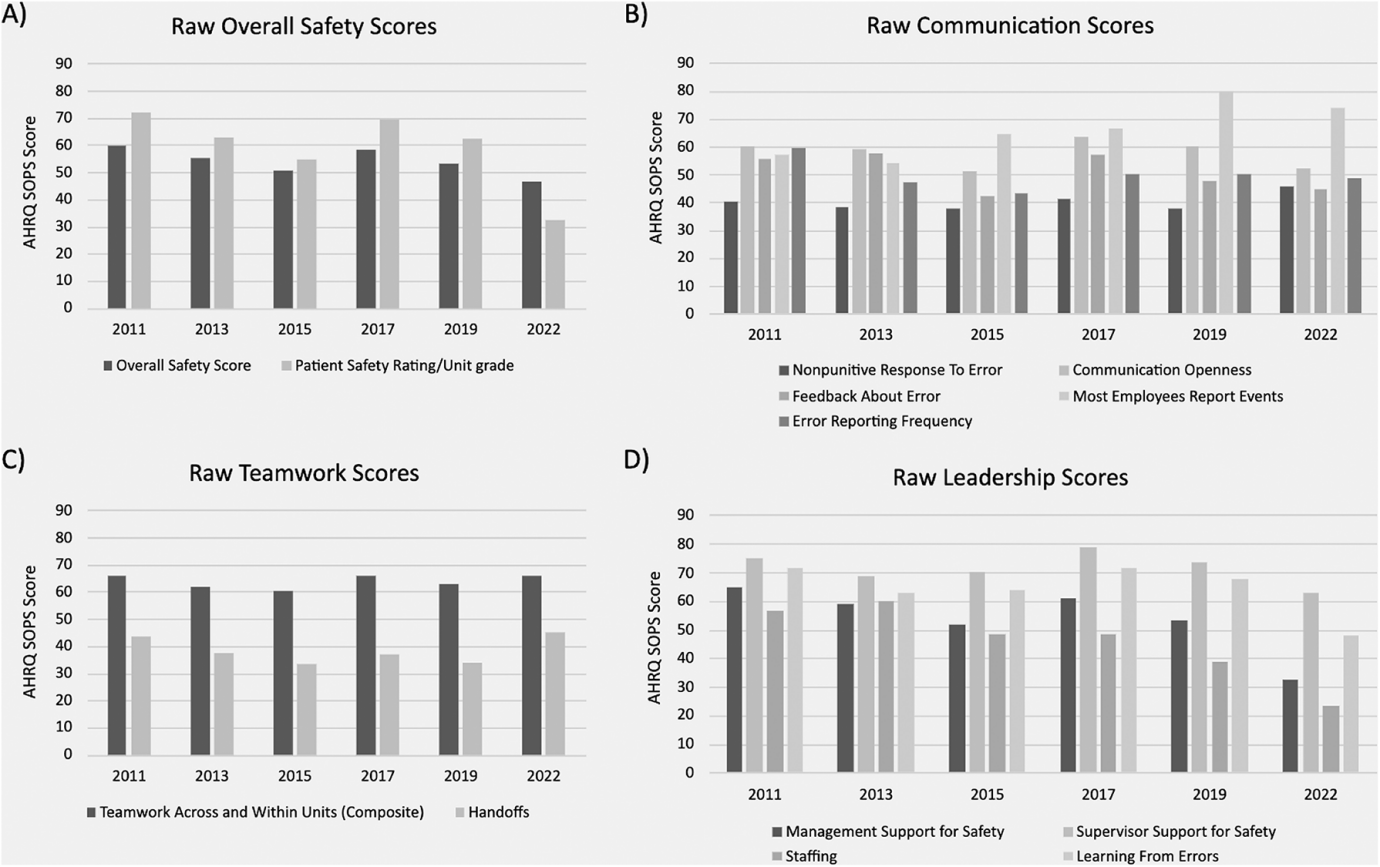
Default AHRQ SOPS results presentation – positive scores only. **(A)** displays raw overall safety scores with patient safety rating/unit grade over time. **(B)** shows the reported frequency and types of communication observed over time. **(C)** displays reported teamwork scores across/within units vs during handoffs over time. **(D)** shows reported leadership scores appreciated over time.

**Figure 2:**
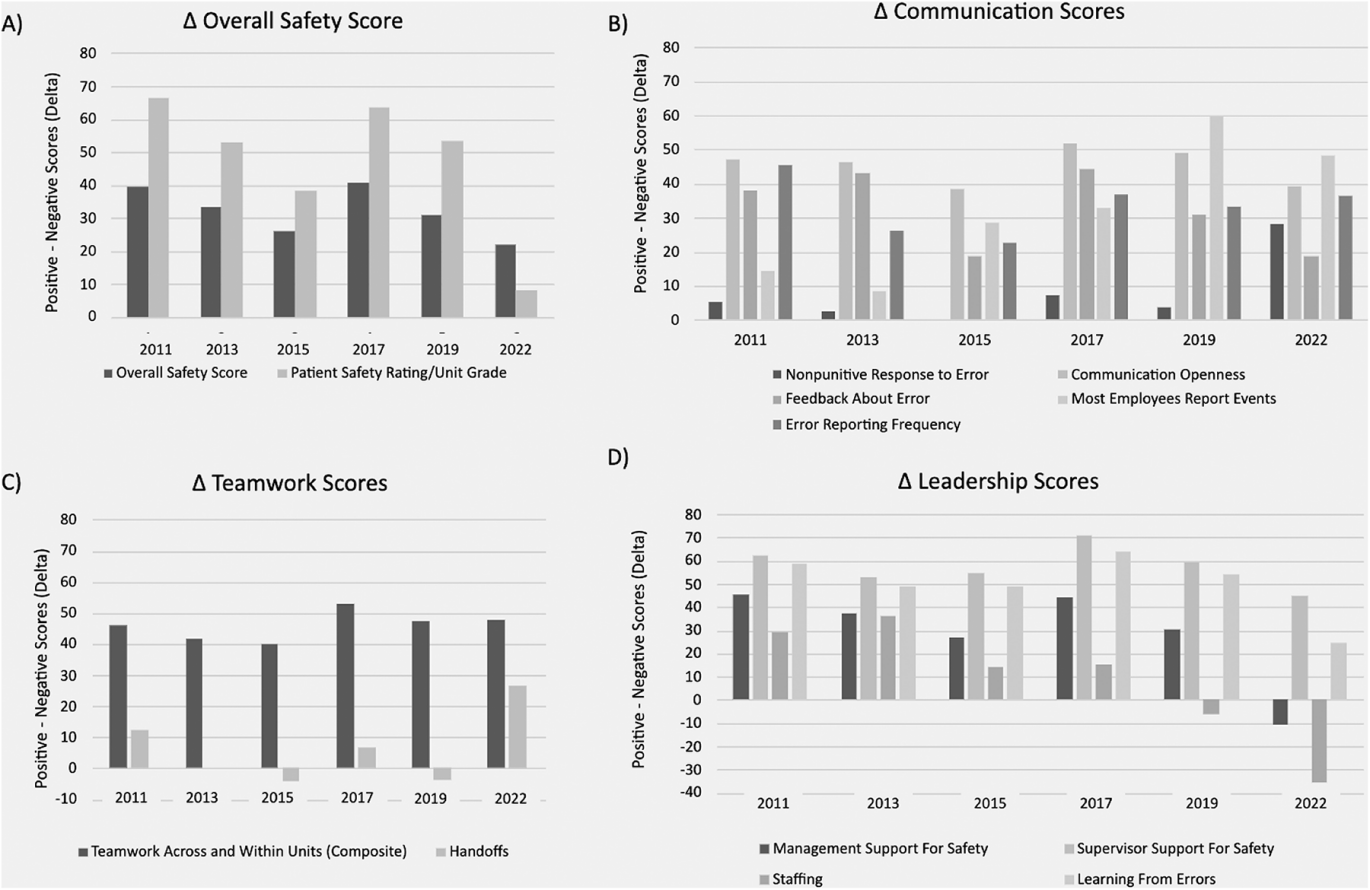
Plotting of AHRQ delta values (% positive responses – % negative responses). **(A–D)** display the change in overall safety, communication, teamwork, and leadership scores, respectively, when accounting for the percentage of negative responses.

Plots of the positive (% positive) and Delta (% positive—negative) scores are displayed in Figures 1, 2, respectively. In Figure 1, *Panel A,* the plot of positive scores demonstrates a gradual decline and weak effect size in both the “Overall Safety Score” and “Patient Safety Rating/Unit Grade”. The plot of Delta values in Figure 2, *Panel A* shows similar declines. *Panel B* in both Figures 1, 2 indicates that under the “Communication” category, the score for ‘Most Employees Report Events” was the only score to gradually increase over time with a strong effect size, while the “Nonpunitive Response to Error” demonstrated an increase in Delta values with a weak effect size, which was the only detected differences between the two plots and *R*^2^ values. *Panel C* in Figure 2 demonstrates the “Handoff” scores on the Delta plot dipped below a score of 0 in 2015 and 2019 indicating more negative than positive responses. Lastly, in both figures, *Panel D* highlights that under “Leadership”, the scores for ‘Management Support for Safety” and “Staffing” demonstrated the most decline of all scores, with moderate and strong effect sizes, respectively. The “Staffing” scores on the Delta plot dipped below a score of 0 in 2019 and 2022 indicating more negative than positive responses.

## Discussion

Overall, the longitudinal analysis of the SOPS over an 11-year period within surgical departments provides valuable insights into the changing dynamics of safety culture. This study employed an innovative approach by considering both the percentage of positive responses and the calculated Delta values, shedding light on nuanced shifts in perception over time. The results from both analyses are similar but not the exact same; this suggests that the most concerning declines over time are in the perception of the patient safety rating at the unit-specific level, top management support for safety, and staffing levels. Similar findings have been reported by recent studies that found healthcare workers displayed a decrease in the perception of patient safety across most dimensions. Some of the contributory factors were lack of staffing, hospital management support, and organizational learning (4, 16–19). Another article found that staffing challenges characterized by extended working hours, high turnover rates, and dissatisfaction with workload and salaries also led to a decrease in perceived patient safety. These findings have been exacerbated by the COVID-19 pandemic due to increased employee workload and stress, shifts in priorities, fear of repercussions, communication challenges, and staffing changes (20–22).

The results are more easily notable in the analysis of the Delta scores (Figure 2), where all three mentioned categories neared or dipped below a score of zero. By calculating the Delta scores for each category, a more thorough and visual understanding of the survey results can be appreciated. For example, in Figure 2, the patient safety rating at the unit level in the year 2022 can be seen to decline to near zero (vs. a score of 32.2% positive responses in Figure 1), which indicates that the proportion of positive and negative responses is almost equal, representing a divergent pattern. This suggests that if left uncorrected, it may lead to a crossing pattern where most respondents now report negative perceptions. Similarly, using the same analogy, the crossing patterns can be noted for the top management support for safety and staffing categories. The comparison for Delta scores to positive-only scores highlighted a change in effect size for “Nonpunitive Response to Error”. This shift, from a very weak effect to a moderate effect, emphasizes the importance of considering both positive and negative responses in comprehensively understanding changes in safety culture.

The examination of overall safety culture positive scores revealed distinctive trends. The “Overall Safety Score” and “Patient Safety Rating/Unit Grade’ exhibited a weak trend with a negative effect size, suggesting a gradual decline in the perception of safety over the studied period. Similarly, “Management Support for Safety” demonstrated a negative trend with a moderate effect size, indicating a noteworthy decrease in positive sentiments regarding management support. The Leadership subcategory scores for “Staffing” and “Learning from Errors” exhibited a negative trend with a strong effect size, reflecting substantial declines in perceptions over time. Conversely, the “Communication” category specifically “Most Employees Report Events”, displayed a positive trend with a strong effect size, indicating a notable improvement in communication-related aspects of safety culture.

Interestingly, taking all the results in conjunction, the scores under the broader and specific categories remain relatively stable or convergent for 2011–2022, suggesting that despite the perceived decline in patient safety rating at the local level, top leadership support and staffing, the individual healthcare teams are relatively unchanged in the way they interact and perceive their roles, communication, and teamwork. This offers a potential narrative highlighting the grit and resilience of the surgical department workforce, especially given the challenges of the COVID-19 pandemic on the healthcare system (e.g., burnout, moral distress, trauma). Declines in patient safety ratings at the local level, top leadership support, and staffing can be partially associated with the COVID-19 pandemic (e.g., non-clinical leaders working from home, physicians and nurses leaving the profession, constant changes to clinical and operational protocols, less opportunity to learn from errors due to high workloads). Similar conclusions were noted by other studies conducted during the COVID-19 pandemic (23–25). One noticeable result from our study was that executive hospital leadership, while having high expectations from the healthcare workforce, was perceived to suboptimally provide the necessary support and empowerment that is crucial for maintaining patient safety and overall well-being of healthcare workers.

It is essential to address some inherent limitations that can influence the generalizability and applications of the data trends. First, the sample size, while met the minimum requirements for analysis per SOPS recommendations (13) comprised of data collected from surgical departments at a single academic institution is relatively limited and varier by year. We also recognize that the matching of questions between SOPS 1.0 and 2.0 is not trivial and could introduce some level of uncertainty in the combined analysis. While the dataset provides interesting insights into patient safety culture within surgical departments, it may not represent the diversity and complexity in healthcare nationally or globally. The dynamics and behaviors of patient safety culture can vary between institutions, regions, and specialties in healthcare. As a result, caution should be exercised when generalizing these results to other healthcare organizations. Second, even with a larger sample size, the study's exclusive focus on a single institution raises questions about using these results to create interventions that nurture a more cohesive patient safety culture in different healthcare settings. Other organizations vary widely in size, governance, and operational practices, and these differences can profoundly impact their patient safety culture. Lastly, it is important to mention that none of the improvement efforts developed and implemented through the course of this longitudinal project was considered or factored into our analysis. This serves as another limitation as it serves as a lead point for bias that can skew results.

The findings in this study lay the foundations for several other avenues of future research aimed at enhancing our understanding of patient safety culture within healthcare organizations and developing more effective interventions. Building on the methodology employed in this study, future research could delve deeper into the dynamics of patient safety culture by conducting Delta analyses across different organizational roles within healthcare settings. For instance, separate analyses could be performed for top management, local management, physicians, nurses, and other healthcare professionals. This granular approach can reveal nuanced variations in how different groups perceive and experience patient safety culture. Understanding each role's specific concerns, strengths, and weaknesses can guide targeted interventions and improvements. This data could offer a more comprehensive view of cultural trends within institutions. The current study, while limited to one academic institution, emphasizes the need for broader investigations across a more extensive range of healthcare organizations. Future studies should consider the incorporation of larger and more diverse datasets from other healthcare settings (i.e., hospitals, clinics, etc.). By including data from different healthcare systems and regions, researchers can identify commonalities and distinctions in trends of patient safety culture. Moreover, large-scale studies have the potential to identify overarching patterns over time.

## Conclusions

Overall, this longitudinal study of SOPS within surgical departments over an 11-year period offers valuable insights into the evolving dynamics of patient safety culture at a large academic institution. By utilizing an innovative approach that considers both positive and negative responses through Delta scores and analyzing longitudinal patterns, the study reveals important shifts in perceptions that may not be captured by traditional methods focused solely on the percentage of positive response rates. The findings indicate that while certain aspects of safety culture, such as communication and reporting, have shown improvement over time, notable declines were observed in areas critical to patient safety, including “Management Support for Safety”, “Staffing”, and the overall “Patient Safety Rating”. The Delta scores highlight these concerning trends more clearly, particularly where positive and negative responses have converged or crossed, signaling areas in need of further exploration.

## Data Availability

The original contributions presented in the study are included in the article/Supplementary Material, further inquiries can be directed to the corresponding author.
